# Retracted: Analysis of China's Population Flow between Urban and Rural Areas and the Reform of Public Health Old-Age Insurance System under the Background of Sustainable Ecological Environment

**DOI:** 10.1155/2023/9853235

**Published:** 2023-02-05

**Authors:** Journal of Environmental and Public Health

*Journal of Environmental and Public Health* has retracted the article titled “Analysis of China's Population Flow between Urban and Rural Areas and the Reform of Public Health Old-Age Insurance System under the Background of Sustainable Ecological Environment” [1] due to concerns that the peer review process has been compromised.

Following an investigation conducted by the Hindawi Research Integrity team [2], significant concerns were identified with the peer reviewers assigned to this article; the investigation has concluded that the peer review process was compromised. We therefore can no longer trust the peer review process, and the article is being retracted with the agreement of the Chief Editor.
